# Supplementary material for: Dynamics of care and sector use between birth, contraception and sick child services
Source: PLOS Glob Public Health. 2025 May 7;5(5):e0004418. doi: 10.1371/journal.pgph.0004418 (PMC12057983; doi:10.1371/journal.pgph.0004418)
Supplement: Table A in S1 Tables — Note: AOR: adjusted odds ratio; CI: confidence interval; Ref: reference; ANC: antenatal care. C-section: cesarean birth. (DOCX) [file pgph.0004418.s001.docx]

**S1 Tables: Supplemental Tables**

Table A. Adjusted odds ratios and 95% confidence intervals for having a missed opportunity for use of modern contraceptives, comparing sectors and use of health services for birth and family planning among women with a need for birth, family planning, or sick child services

|  | **Afghanistan 2015** | | **India 2015-16** | | **Indonesia 2017** | | **Kenya 2014** | |
| --- | --- | --- | --- | --- | --- | --- | --- | --- |
|  | **AOR (p-value)** | **95% CI** | **AOR (p-value)** | **95% CI** | **AOR (p-value)** | **95% CI** | **AOR (p-value)** | **95% CI** |
| **Delivery Source** |  |  |  |  |  |  |  |  |
| Private | 0.54 (0.014) | [0.33,0.88] | 1.23 (0.000) | [1.12,1.36] | 1.27 (0.028) | [1.03,1.56] | 0.86 (0.567) | [0.52,1.44] |
| Other/None | 1.06 (0.619) | [0.85,1.31] | 1.17 (0.010) | [1.04,1.32] | 1.38 (0.018) | [1.06,1.80] | 1.53 (0.010) | [1.11,2.11] |
| **Place of residence (ref = Urban)** | | | | | | | | |
| Rural | 1.03 (0.873) | [0.72,1.46] | 0.99 (0.869) | [0.89,1.10] | 0.67 (0.000) | [0.55,0.81] | 1.51 (0.012) | [1.10,2.09] |
| **Wealth(ref = Low)** |  |  |  |  |  |  |  |  |
| Medium | 1.10 (0.527) | [0.81,1.50] | 0.90 (0.023) | [0.82,0.99] | 1.19 (0.157) | [0.94,1.50] | 0.51 (0.000) | [0.37,0.71] |
| High | 0.71 (0.072) | [0.49,1.03] | 0.82 (0.001) | [0.74,0.92] | 1.41 (0.009) | [1.09,1.83] | 0.40 (0.000) | [0.27,0.61] |
| **Education (ref = none or primary)** | | | | | | | | |
| Secondary or higher | 0.61 (0.025) | [0.39,0.94] | 0.97 (0.492) | [0.90,1.05] | 1.41 (0.003) | [1.12,1.76] | 0.90 (0.512) | [0.65,1.24] |
| **Parity(ref = 2+ children)** | | | | | | | | |
| 1 child | 1.78 (0.001) | [1.26,2.51] | 2.44 (0.000) | [2.26,2.64] | 1.23 (0.023) | [1.03,1.47] | 1.56 (0.036) | [1.03,2.35] |
| **Number of ANC visits (ref = 0-3 visits)** | | | | | | | | |
| 4 or more ANC visits | 0.83 (0.190) | [0.64,1.09] | 0.89 (0.003) | [0.82,0.96] | 0.72 (0.020) | [0.55,0.95] | 0.61 (0.001) | [0.45,0.82] |
| **C-section (ref = No)** |  |  |  |  |  |  |  |  |
| Yes | 0.55 (0.144) | [0.24,1.23] | 0.72 (0.000) | [0.66,0.79] | 0.86 (0.166) | [0.69,1.07] | 1.07 (0.849) | [0.52,2.23] |
| **Any problem accessing care (ref = No)** | | | | | | | | |
| Yes | 1.12 (0.651) | [0.68,1.84] | 1.01 (0.784) | [0.94,1.08] | 1.04 (0.692) | [0.87,1.24] | 0.96 (0.751) | [0.73,1.25] |
|  |  |  |  |  |  |  |  |  |
|  | **Malawi 2015-16** | | **Nigeria 2018** | | **Pakistan 2017-2018** | | **Uganda 2016** | |
|  | **AOR(p-value)** | **95% CI** | **AOR(p-value)** | **95% CI** | **AOR(p-value)** | **95% CI** | **AOR(p-value)** | **95% CI** |
| **Delivery Source (ref = Public)** | | | | | | | | |
| Private | 1.11 (0.395) | [0.88,1.40] | 1.68 (0.004) | [1.18,2.39] | 1.21 (0.197) | [0.91,1.62] | 1.09 (0.549) | [0.82,1.45] |
| Other/None | 1.22 (0.188) | [0.91,1.64] | 1.37 (0.014) | [1.07,1.76] | 1.46 (0.024) | [1.05,2.04] | 1.24 (0.028) | [1.02,1.50] |
| **Place of residence (ref = Urban)** | | | | | | | | |
| Rural | 1.42 (0.090) | [0.95,2.14] | 0.87 (0.337) | [0.66,1.16] | 0.86 (0.321) | [0.64,1.16] | 1.42 (0.015) | [1.07,1.87] |
| **Wealth (ref = Low)** |  |  |  |  |  |  |  |  |
| Medium | 0.91 (0.393) | [0.74,1.13] | 0.86 (0.332) | [0.63,1.17] | 0.76 (0.101) | [0.55,1.06] | 0.68 (0.000) | [0.55,0.83] |
| High | 0.88 (0.381) | [0.67,1.16] | 0.56 (0.002) | [0.39,0.82] | 0.63 (0.036) | [0.41,0.97] | 0.53 (0.000) | [0.41,0.69] |
| **Education (ref = none or primary)** | | | | | | | | |
| Secondary or higher | 0.90 (0.463) | [0.68,1.19] | 0.52 (0.000) | [0.39,0.69] | 1.12 (0.452) | [0.84,1.50] | 0.79 (0.038) | [0.63,0.99] |
| **Parity (ref = 2+ children)** | |  |  |  |  |  |  |  |
| 1 child | 0.78 (0.037) | [0.62,0.99] | 1.18 (0.378) | [0.82,1.70] | 1.93 (0.000) | [1.38,2.69] | 1.06 (0.664) | [0.81,1.39] |
| **Number of ANC visits (ref = 0-3 visits)** | | | | | | | | |
| 4 or more ANC visits | 0.83 (0.036) | [0.70,0.99] | 0.51 (0.000) | [0.40,0.65] | 0.98 (0.883) | [0.75,1.28] | 0.96 (0.685) | [0.81,1.15] |
| **C-section(ref = No)** |  |  |  |  |  |  |  |  |
| Yes | 0.89 (0.567) | [0.59,1.34] | 0.66 (0.110) | [0.39,1.10] | 0.58 (0.001) | [0.42,0.81] | 0.88 (0.505) | [0.59,1.29] |
| **Any problem accessing care (ref = No)** | | | | | | | | |
| Yes | 0.95 (0.618) | [0.78,1.16] | 1.26 (0.037) | [1.01,1.58] | 1.07 (0.649) | [0.81,1.41] | 1.11 (0.298) | [0.91,1.36] |
| Note: AOR: adjusted odds ratio; CI: confidence interval; Ref: reference; ANC: antenatal care. C-section: cesarean birth. | | | | | | | | |

Table B. Adjusted odds ratios and 95% confidence intervals for having missed oppportunity for care for sick children, comparing sectors and use of a facility for birth among women with a need for birth, family planning, or a sick child services

|  | **Afghanistan 2015** | | **India 2015-16** | | **Indonesia 2017** | | **Kenya 2014** | |
| --- | --- | --- | --- | --- | --- | --- | --- | --- |
|  | **AOR (p-value)** | **95% CI** | **AOR (p-value)** | **95% CI** | **AOR (p-value)** | **95% CI** | **AOR (p-value)** | **95% CI** |
| **Delivery Source (ref = Public)** | | | | | | | | |
| Private | 0.75(0.282) | [0.45,1.26] | 0.88 (0.011) | [0.80,0.97] | 1.11 (0.343) | [0.90,1.36] | 1.34 (0.136) | [0.91,1.96] |
| Other/None | 1.34 (0.028) | [1.03,1.73] | 1.11 (0.070) | [0.99,1.24] | 1.07 (0.626) | [0.82,1.39] | 1.55 (0.003) | [1.17,2.06] |
| **Place of residence (ref = Urban)** | | | | | | | | |
| Rural | 0.99 (0.928) | [0.72,1.36] | 1.01 (0.869) | [0.91,1.12] | 0.99 (0.904) | [0.81,1.21] | 0.78 (0.072) | [0.60,1.02] |
| **Wealth (ref = Low)** |  |  |  |  |  |  |  |  |
| Medium | 0.90 (0.380) | [0.70,1.15] | 0.89 (0.011) | [0.81,0.97] | 0.69 (0.001) | [0.55,0.85] | 0.88 (0.379) | [0.66,1.17] |
| High | 0.82 (0.250) | [0.58,1.15] | 0.83 (0.002) | [0.74,0.93] | 0.73 (0.012) | [0.57,0.93] | 0.76 (0.107) | [0.54,1.06] |
| **Education (ref = none or primary)** | | | | | | | | |
| Secondary or higher | 0.76 (0.285) | [0.45,1.26] | 0.95 (0.220) | [0.87,1.03] | 1.22 (0.078) | [0.98,1.53] | 0.97 (0.824) | [0.71,1.32] |
| **Parity (ref = 2+ children)** | | | | | | | | |
| 1 child | 0.80 (0.159) | [0.58,1.09] | 0.91 (0.022) | [0.83,0.99] | 1.02 (0.842) | [0.84,1.24] | 1.24 (0.238) | [0.87,1.79] |
| **Number of ANC visits (ref = 0-3 visits)** | | | | | | | | |
| 4 or more ANC visits | 0.70 (0.018) | [0.53,0.94] | 0.88 (0.002) | [0.82,0.96] | 0.68 (0.004) | [0.52,0.88] | 0.57 (0.000) | [0.44,0.73] |
| **C-section (ref = No)** | | | | | | | | |
| Yes | 1.14 (0.635) | [0.66,1.97] | 0.96 (0.405) | [0.87,1.06] | 1.00 (1.000) | [0.78,1.28] | 1.04 (0.896) | [0.59,1.82] |
| **Any problem accessing care (ref = No)** | | | | | | | | |
| Yes | 0.97 (0.890) | [0.64,1.48] | 1.18 (0.000) | [1.09,1.27] | 1.30 (0.004) | [1.09,1.55] | 0.90 (0.449) | [0.69,1.18] |
|  |  |  |  |  |  |  |  |  |
|  | **Malawi 2015-16** | | **Nigeria 2018** | | **Pakistan 2017-2018** | | **Uganda 2016** | |
|  | **AOR (p-value)** | **95% CI** | **AOR (p-value)** | **95% CI** | **AOR (p-value)** | **95% CI** | **AOR (p-value)** | **95% CI** |
| **Delivery Source (ref = Public)** | | | | | | | | |
| Private | 1.08 (0.520) | [0.85,1.39] | 1.37 (0.085) | [0.96,1.95] | 1.15 (0.379) | [0.84,1.59] | 0.93 (0.653) | [0.69,1.26] |
| Other/None | 1.64 (0.001) | [1.23,2.18] | 1.36 (0.009) | [1.08,1.72] | 1.11 (0.576) | [0.77,1.60] | 1.03 (0.787) | [0.81,1.32] |
| **Place of residence (ref = Urban)** | | | | | | | | |
| Rural | 0.66 (0.003) | [0.50,0.87] | 1.08 (0.522) | [0.85,1.37] | 1.18 (0.243) | [0.89,1.55] | 0.97 (0.840) | [0.69,1.35] |
| **Wealth (ref = Low)** |  |  |  |  |  |  |  |  |
| Medium | 1.00 (0.967) | [0.82,1.23] | 0.81 (0.111) | [0.63,1.05] | 0.86 (0.410) | [0.61,1.23] | 0.84 (0.158) | [0.66,1.07] |
| High | 1.06 (0.600) | [0.86,1.30] | 0.64 (0.007) | [0.46,0.88] | 0.89 (0.608) | [0.58,1.37] | 0.73 (0.038) | [0.54,0.98] |
| **Education (ref = none or primary)** | | | | | | | | |
| Secondary or higher | 0.86 (0.154) | [0.69,1.06] | 0.88 (0.320) | [0.68,1.14] | 0.95 (0.773) | [0.68,1.33] | 0.84 (0.151) | [0.66,1.07] |
| **Parity (ref = 2+ children)** | |  |  |  |  |  |  |  |
| 1 child | 0.96 (0.646) | [0.80,1.15] | 0.99 (0.933) | [0.70,1.39] | 0.72 (0.138) | [0.47,1.11] | 1.24 (0.109) | [0.95,1.61] |
| **Number of ANC visits (ref = 0-3 visits)** | | | | | | | | |
| 4 or more ANC visits | 0.88 (0.089) | [0.76,1.02] | 0.69 (0.001) | [0.56,0.86] | 0.78 (0.092) | [0.58,1.04] | 0.78 (0.011) | [0.64,0.94] |
| **C-section (ref = No)** | | | | | | | | |
| Yes | 0.79 (0.149) | [0.58,1.09] | 1.00 (1.000) | [0.55,1.82] | 1.12 (0.546) | [0.78,1.62] | 1.22 (0.264) | [0.86,1.75] |
| **Any problem accessing care (ref = No)** | | | | | | | | |
| Yes | 1.22 (0.016) | [1.04,1.44] | 1.29 (0.017) | [1.05,1.58] | 1.12 (0.426) | [0.84,1.50] | 1.04 (0.682) | [0.86,1.26] |
| Note: AOR: adjusted odds ratio; CI: confidence interval; Ref: reference; ANC: antenatal care. C-section: cesarean birth. | | | | | | | | |

Table C. Adjusted odds ratios and 95% confidence intervals for having missed care for sick children, comparing sectors and use of health services for birth and family planning among women with a need for birth, family planning, or sick child services

|  | **Afghanistan 2015** | | **India 2015-16** | | **Indonesia 2017** | | **Kenya 2014** | |
| --- | --- | --- | --- | --- | --- | --- | --- | --- |
|  | **AOR (p-value)** | **95% CI** | **AOR (p-value)** | **95% CI** | **AOR (p-value)** | **95% CI** | **AOR (p-value)** | **95% CI** |
| **Sector use (ref = Sector fidelity)** | | | | | | | | |
| Switched sectors | 1.08 (0.708) | [0.71,1.65] | 0.93 (0.228) | [0.82,1.05] | 0.95 (0.722) | [0.74,1.23] | 1.10 (0.622) | [0.75,1.63] |
| Facility birth to no FP | 1.42 (0.046) | [1.01,2.01] | 0.99 (0.833) | [0.90,1.08] | 1.42 (0.006) | [1.11,1.83] | 1.03 (0.902) | [0.67,1.59] |
| No facility birth to FP | 1.29 (0.226) | [0.85,1.94] | 1.14 (0.130) | [0.96,1.34] | 0.98 (0.896) | [0.74,1.30] | 1.36 (0.076) | [0.97,1.92] |
| No facility birth and no FP | 2.04 (0.000) | [1.37,3.05] | 1.09 (0.277) | [0.93,1.28] | 1.47 (0.086) | [0.95,2.28] | 1.85 (0.002) | [1.26,2.71] |
| **Place of residence (ref = Urban)** | | | | | | | | |
| Rural | 1.00 (0.993) | [0.72,1.38] | 1.01 (0.829) | [0.91,1.12] | 1.00 (0.983) | [0.82,1.22] | 0.77 (0.055) | [0.59,1.01] |
| **Wealth (ref = Low)** |  |  |  |  |  |  |  |  |
| Medium | 0.89 (0.360) | [0.70,1.14] | 0.88 (0.009) | [0.81,0.97] | 0.68 (0.000) | [0.55,0.84] | 0.90 (0.450) | [0.68,1.19] |
| High | 0.84 (0.305) | [0.59,1.18] | 0.82 (0.000) | [0.73,0.92] | 0.72 (0.011) | [0.56,0.93] | 0.79 (0.184) | [0.57,1.12] |
| **Education (ref = none or primary)** | | | | | | | | |
| Secondary or higher | 0.77 (0.313) | [0.46,1.28] | 0.95 (0.189) | [0.87,1.03] | 1.20 (0.104) | [0.96,1.50] | 0.99 (0.964) | [0.73,1.35] |
| **Parity (ref = 2+ children)** |  |  |  |  |  |  |  |  |
| 1 child | 0.77 (0.101) | [0.56,1.05] | 0.91 (0.027) | [0.83,0.99] | 1.01 (0.894) | [0.84,1.23] | 1.22 (0.284) | [0.85,1.77] |
| **Number of ANC visits (ref = 0-3 visits)** | | | | | | | | |
| 4 or more ANC visits | 0.71 (0.021) | [0.54,0.95] | 0.88 (0.002) | [0.82,0.95] | 0.69 (0.006) | [0.53,0.90] | 0.58 (0.000) | [0.45,0.74] |
| **C-section (ref = No)** | | | | | | | | |
| Yes | 1.17 (0.551) | [0.69,1.99] | 0.92 (0.086) | [0.84,1.01] | 1.00 (0.990) | [0.78,1.28] | 1.05 (0.871) | [0.60,1.85] |
| **Any problem accessing care (ref = No)** | | | | | | | | |
| Yes | 0.97 (0.881) | [0.64,1.47] | 1.18 (0.000) | [1.09,1.27] | 1.29 (0.005) | [1.08,1.54] | 0.90 (0.442) | [0.69,1.18] |
|  |  |  |  |  |  |  |  |  |
|  | **Malawi 2015-16** | | **Nigeria 2018** | | **Pakistan 2017-2018** | | **Uganda 2016** | |
|  | **AOR (p-value)** | **95% CI** | **AOR (p-value)** | **95% CI** | **AOR (p-value)** | **95% CI** | **AOR (p-value)** | **95% CI** |
| **Sector use (ref = Sector fidelity)** | |  |  |  |  |  |  |  |
| Switched sectors | 1.18 (0.164) | [0.93,1.50] | 0.57 (0.046) | [0.33,0.99] | 1.72 (0.033) | [1.05,2.84] | 1.22 (0.208) | [0.89,1.68] |
| Facility birth to no FP | 1.25 (0.031) | [1.02,1.52] | 1.19 (0.381) | [0.81,1.75] | 1.54 (0.061) | [0.98,2.41] | 1.50 (0.001) | [1.18,1.90] |
| No facility birth to FP | 1.56 (0.008) | [1.12,2.18] | 1.14 (0.553) | [0.74,1.76] | 1.27 (0.355) | [0.76,2.13] | 1.02 (0.883) | [0.74,1.41] |
| No facility birth and no FP | 2.46 (0.001) | [1.43,4.22] | 1.47 (0.056) | [0.99,2.19] | 1.71 (0.042) | [1.02,2.86] | 1.65 (0.004) | [1.17,2.32] |
| **Place of residence (ref = Urban)** | |  |  |  |  |  |  |  |
| Rural | 0.67 (0.005) | [0.51,0.88] | 1.09 (0.474) | [0.86,1.39] | 1.19 (0.219) | [0.90,1.57] | 0.94 (0.700) | [0.67,1.31] |
| **Wealth (ref = Low)** |  |  |  |  |  |  |  |  |
| Medium | 1.01 (0.949) | [0.82,1.23] | 0.81 (0.115) | [0.63,1.05] | 0.87 (0.456) | [0.61,1.25] | 0.87 (0.254) | [0.69,1.11] |
| High | 1.06 (0.589) | [0.86,1.30] | 0.66 (0.015) | [0.48,0.92] | 0.89 (0.596) | [0.57,1.38] | 0.75 (0.059) | [0.56,1.01] |
| **Education (ref = none or primary)** | |  |  |  |  |  |  |  |
| Secondary or higher | 0.85 (0.131) | [0.68,1.05] | 0.91 (0.503) | [0.70,1.19] | 0.94 (0.700) | [0.67,1.31] | 0.85 (0.172) | [0.67,1.07] |
| **Parity (ref = 2+ children)** |  |  |  |  |  |  |  |  |
| 1 child | 0.97 (0.737) | [0.81,1.16] | 0.98 (0.919) | [0.69,1.39] | 0.68 (0.080) | [0.44,1.05] | 1.22 (0.135) | [0.94,1.59] |
| **Number of ANC visits (ref = 0-3 visits)** | |  |  |  |  |  |  |  |
| 4 or more ANC visits | 0.89 (0.113) | [0.77,1.03] | 0.71 (0.002) | [0.57,0.88] | 0.78 (0.093) | [0.58,1.04] | 0.78 (0.013) | [0.65,0.95] |
| **C-section (ref = No)** |  |  |  |  |  |  |  |  |
| Yes | 0.80 (0.159) | [0.58,1.09] | 1.09 (0.790) | [0.59,1.98] | 1.21 (0.313) | [0.83,1.77] | 1.24 (0.246) | [0.86,1.78] |
| **Any problem accessing care (ref = No)** | |  |  |  |  |  |  |  |
| Yes | 1.22 (0.018) | [1.03,1.43] | 1.28 (0.019) | [1.04,1.57] | 1.10 (0.535) | [0.82,1.46] | 1.03 (0.764) | [0.85,1.24] |
| Note: AOR: adjusted odds ratio; CI: confidence interval; Ref: reference; ANC: antenatal care; C-section: cesarean birth. | | | | | | | | |
