# Supplementary material for: Dynamics of care and sector use between birth, contraception and sick child services
Source: PLOS Glob Public Health. 2025 May 7;5(5):e0004418. doi: 10.1371/journal.pgph.0004418 (PMC12057983; doi:10.1371/journal.pgph.0004418)
Supplement: Attachment — Submitted filename: PLOS GPH Response Letter - Sector Use Dynamics_Revsions.docx [file pgph.0004418.s005.docx]

Dear Dr. Tappis,

*Thank you for the opportunity to revise and resubmit our manuscript for consideration for publication in PLOS Global Public Health. We are deeply grateful for the insightful and salient comments provided by the reviewers. We have taken care to address the concerns as best as possible, to be more precise in our language, and to highlight important issues mentioned by reviewers.*

Journal Requirements:

1. Please provide an Author Summary. This should appear in your manuscript between the Abstract (if applicable) and the Introduction, and should be 150–200 words long. The aim should be to make your findings accessible to a wide audience that includes both scientists and non-scientists. Sample summaries can be found on our website under Submission Guidelines: <https://journals.plos.org/globalpublichealth/s/submission-guidelines#loc-parts-of-a-submission>.

*We received communication on 2/27 confirming an Author Summary was not necessary.*

2. Please review your reference list to ensure that it is complete and correct. If you have cited papers that have been retracted, please include the rationale for doing so in the manuscript text, or remove these references and replace them with relevant current references. Any changes to the reference list should be mentioned in the rebuttal letter that accompanies your revised manuscript. If you need to cite a retracted article, indicate the article’s retracted status in the References list and also include a citation and full reference for the retraction notice.

*We have not identified any retracted articles.*

Reviewer #1: Feedback on the Manuscript – Introduction and Method Section Overall Impression:

Introduction: The introduction provides a strong foundation for the study, effectively outlining the importance of understanding patterns of care seeking across the public and private sectors for family planning, reproductive health, and maternal, newborn, and child health services.

*Thank you for the positive and constructive feedback.*

Specific Feedback:

1. Strengthen the Thesis Statement: While the introduction provides a clear overview, consider adding a more focused thesis statement that directly states the research question or objective of the study. For example, "This study aims to examine patterns of sector use and sector fidelity for childbirth, modern contraceptive use, and sick child care among women in eight countries in Asia and sub-Saharan Africa."

*Thank you. We detailed our research aims at the end of the Introduction sections and have revised the text to strengthen this section. It reads:*

*“To address the gaps in the literature, we analyzed Demographic and Health Survey (DHS) data to examine sector use and sector fidelity at the individual level for childbirth, modern contraceptives, and sick child care across eight countries in Asia and sub-Saharan Africa.*

*The three research questions addressed by these analyses are as follows:*

1. *What are the patterns of sector use, sector switching, and missed opportunities for care across childbirth, modern contraceptives, and sick child care*
2. *Is sector for birth associated with subsequent missed opportunities for using modern contraceptives and seeking sick child care?*
3. *Are sector fidelity and switching sectors across birth and contraception associated with subsequent missed opportunities for sick child care?*
2. Discuss the Implications of Sector Switching: Elaborate on the potential implications of sector switching for continuity of care and health outcomes. For example, you could discuss the risks of missed opportunities for follow-up care or the potential for fragmented care.

*We elaborated on our previous passage (p. 3) on this and revised the text under Reviewer #2’s suggestion. This passage now reads, “The reasons for switching sectors are likely highly individualized and context-specific, and there is a dearth of research exploring this. For example, individuals may switch sectors because they prefer a certain provider, or because they have been referred to a provider in a different sector (and have the financial and transportation means to reach that provider). Conversely, sector switching could indicate a discontinuity of care or fragmented care—due to negative experiences, inadequate resources, or lack of options for continuing care in the same sector. These experiences may hinder future healthcare seeking, leading to missed opportunities for follow-up and referrals and potentially worsening health outcomes if care is delayed or not accessed. Though sector fidelity does not guarantee continuity of care, it may also facilitate future care seeking and result in fewer missed opportunities.*

3. Highlight the Study's Contribution: Explain how the study will contribute to the existing literature on public-private health systems and women's health.

*Following the passage above, our paper highlights “However, the dynamics and implications of health seeking behavior across sectors are not well understood. An improved understanding of these patterns of care seeking can inform strategic communication interventions and guide FP and MNCH investments across health systems for maximum impact. To address the gaps in the literature, we analyzed Demographic and Health Survey (DHS) data to …”*

Results, Discussion and Conclusion Section Overall Impression: The results section provides a comprehensive overview of the study's findings, effectively presenting the patterns of sector use, sector switching, and missed opportunities for care. The use of tables and figures is helpful in visualizing the data.

*Thank you for these comments and noting the tables and figures helped with visualization.*

Specific Feedback:

1. Discuss the Implications of Sector Switching: While you've identified that sector switching is common, discuss the potential implications of this for continuity of care and health outcomes. For example, explore whether sector switching is associated with better or worse health outcomes compared to sector fidelity.

*We agree that this is an important question; however, this was not one of our stated research aims. Since little was known about sector use, fidelity, and switching across various sectors, our study sought to establish a baseline understanding of the dynamic. Our analysis showed that more than sector switching, nonuse of facilities in the past was the strongest predictor of missed sick child care, which has implications for child health outcomes. To this point, we added: “Our analysis highlights that more important than the sector used or whether switching occurred, previous non-use of health facilities was a predictor of missed care. Ensuring access to high-quality health care, no matter the sector, must remain a priority for improving health outcomes. These findings underscore the need for health system stewards to account for both the public and private sectors in health governance. Greater cross-sectoral cooperation and continuity of care are paramount to improving health outcomes.”*

2. Explore the Role of Quality of Care: Consider exploring the role of quality of care within the public and private sectors in influencing women's decisions to switch sectors or remain with a particular sector.

*We elaborated on our discussion and the paragraph on quality on page 18 now reads as follows: “Both the public and private sectors can more effectively shape future care-seeking behavior at these critical gateway moments by ensuring consistently high-quality, person-centered care. Although the experience and quality of care vary across settings, with no sector consistently outperforming the other across countries, quality of care plays an important role in individuals’ decision-making about choice and sector of facility [35]. Experiencing poor-quality care could understandably deter individuals from seeking future care [3,35]. However, significant improvements are needed in the quality of care across both sectors along the FP and MNCH care continuum [36–38]. Increasingly, digital health tools may also serve to better foster continuity of care and improved patient outcomes for women who drop out or never engage with the health system [39].*

3. Acknowledge Limitations of the Data: Acknowledge potential limitations of the DHS data, such as the reliance on self- reported information or the possibility of recall bias.

*Reviewer #2 had a similar comment, and we have revised our limitations sections to read, “As these surveys were administered to women, and our sample included only women with a birth in the five years preceding the survey, other caretakers may have taken the sick child for care, which the mother may not have reported. Like much research based on self-report survey data, recall and social desirability bias are likely to be present. Although questions about health service use, such as facility-based care and treatment for sick children, are relatively accurately reported [44,45] the ability of respondents to understand, classify, and recall the sector of service used may be limited [46].”*

4. Discuss Policy Implications: Explore the potential policy implications of the findings, such as recommendations for improving the coordination between public and private health systems or for investing in the quality of care in both sectors.

*We are reluctant to provide specific policy recommendations given the need for context-specific considerations, although we note :* “*Global strategies have called for greater recognition and leverage of the private sector within mixed health systems as essential to achieving national and Sustainable Development Goals [26]. Global agencies (such as donors) and governments seeking to leverage the private health sector in LMICs for improved health outcomes should strive to ensure that mechanisms are in place to foster private sector engagement and include these providers in health systems planning and management.”*

*And in our final paragraph we say, “Ideally, any interventions related to sector switching or sector fidelity should first be informed by the local context and an understanding of how service users navigate and interact with the public and private sectors. With greater clarity on how individuals make health care decisions and how mixed health systems are, or are not, serving individuals’ needs, system stewards can improve health outcomes by identifying and addressing gaps that may lead to missed opportunities for care.”*

Reviewer #2: This is an interesting study looking at the use of private vs public sector use for childbirth, family planning and sick child care. I particularly find the "continuum of care" approach refreshing. I find it a bit challenging to lump countries in one big "LMIC" group for this topic as access to care and use of care, especially when it comes to women's health is heavily influenced by local culture. Your results for Afghanistan are interesting-- are you able to find references to explain them? If wonder if the sociopolitical context of the country affect women's ability to access to contraception within the public sector...

*We are grateful to this reviewer for their comments as they have helped strengthen this paper. We agree that it is not appropriate in many contexts to lump all countries together, especially as we show such different patterns of use across countries. The aim of this paper was to describe the patterns we saw across each country as this has not been done before. Given the breadth of countries studied and the differences across settings, and our intention to allow the results to inform country experts to triangulate these findings with their knowledge, we did not see fit to comment on each country in-depth.*

1. line 38. "in use of these three services" sounds awkward, peharps just "pattern in use" will suffice

*We have made this change for readability.*

2. line 47-49-- out of pocket cost and accessibility are not just factors for private sector, also affect use of public sector. are there studies that also look at reputation of public vs private sector as influencing factors?

*This is a good point. We have added:* *Interpersonal and community factors, such as influence from husbands, family members, and neighbors can affect women’s perceptions and decisions about facility use and choice of facility type and sector.[15,16] Other factors relating to these patterns include the types of facilities within the public and private sectors, country policies, the out-of-pocket costs of private sector services, and accessibility of private facilities [17,18].*

3. line 57-62-- I disagree with these statements. Do you have references to support those statements? If not I would consider removing them or rewording them. In my experience, follow ups and referrals are made based on what the patient needs, they are not siloed within each sector. If a public facility does not offer a service, it will refer the patient to a private facility and vice versa-- whether the patient ends up accessing care depends on other factors such as easy access, affordability,... Same for sector fidelity-- does not mean much if patients access care at different facilities each time they need care as their records may not be available from one private facility to the next.

*Thank you for the suggestion to clarify—we were indeed only providing examples that have been reported anecdotally in our and colleagues’ implementation experience. We have reworded the text to say “**Reasons for switching sectors are likely highly individualized and context-specific, and there is a dearth of research exploring this. For example, individuals may switch sectors because they prefer a certain provider, or because they have been referred to a provider in a different sector (and have the financial and transportation means to reach that provider). Conversely, sector switching could indicate a discontinuity of care or fragmented care—due to negative experiences, inadequate resources, or lack of options for continuing care in the same sector. These experiences may hinder future healthcare seeking, leading to missed opportunities for follow-up and referrals and potentially worsening health outcomes if care is delayed or not accessed. Though sector fidelity does not guarantee continuity of care, it may also facilitate future care seeking and result in fewer missed opportunities.*

4. line 61-- why Asia and SSA in particular? line 201-- please insert reference for Stata

*We first selected our countries based on USAID priority status and then further refined the selection based on sample size. We specified this in the Data section and have now made revisions for clarity, “We explored an initial set of 21 United States Agency for International Development Population and Reproductive Health priority countries that have conducted DHS surveys since 2014. Of these 21 countries, we selected the eight countries with the largest possible analytic sample to maximize the reliability and precision of the estimates produced by the analysis. This selection included four countries in sub-Saharan Africa and four in Asia. The eight countries and corresponding year of DHS data collection were Afghanistan (2015), India (2019-21), Indonesia (2017), Kenya (2014), Malawi (2015-16), Nigeria (2018), Pakistan (2017-2018), and Uganda (2016).*

*We have also added a reference for Stata.*

5. line 214-- missing a word "more than ...resided"

*Thank you for catching this. We have revised the text to read: “More than half of the sample in each country resided in rural areas.”*

6. 215-217--unclear which percentages belong to which country line 256-- reads "fig 1". did you mean fig 2?

*We see this was confusing as written and clarified this text to read: “Women in these two countries also demonstrated the lowest and highest percentages of less than four ANC visits (8.5% in Indonesia, 78.2% in Afghanistan) and lowest and highest report of problems accessing care (34.9% in Afghanistan and 88.8% in Afghanistan).*

7. figure 2 -- what does x axis represent?

*The X axis represents the “percent” which is included in the graph label and represents the percent of women with a need for all three services. The title specifies this as “Percentage of women with sector fidelity, sector switching, and missed opportunity for contraception and sick child care, by sectors and use of a facility for birth among women with a need for all three services” We see though that this title in the text is erroneously labeled as Fig 1 and we have made this correction.*

8. lines 321-322-- "Considering the general desirability that women in need of delivery, contraception and sick child health services go on to access these services"-- awkward phrasing, please re-write

*We clarified this to read: “Considering the value of accessing services for birth, contraception, and sick child care, encouragingly, our analysis found that switching between the public and private sectors does not negatively correlate with future health care seeking.”*

9. line 338-- remove the "." before "the findings"

*We replaced the “.” with “,”.*

10. line 344- please insert reference for the Integrated Gateway Model

*We added the reference.*

11. Strengths and limitations-- should mention retrospective nature of data introduces chances of recall bias. Also, sick child care might not be the sole responsibility of the mother-- father or other family member could have taken the child to access care. In some communities, there are community healthcare workers who may be able to take care of minor illnesses-- was that captured in the survey?

*We have revised our limitations sections to incorporate the points related to recall bias and caretaker role to read, “**As these surveys were administered to women, and our sample included only women with a birth in the five years preceding the survey, other caretakers may have taken the sick child for care, which the mother may not have reported. Like much research based on self-reported survey data, recall and social desirability bias are likely to be present. Although questions about health service use, such as facility-based care and treatment for sick children, are relatively accurately reported [44,45] the ability of respondents to understand, classify, and recall the sector of service used may be limited [46].*

*We coded community health workers according to the country-specific health system. These designations are included in the final reports, usually listed in the tables and/or denoted in the questionnaire. We have added this text to the measures section.*
